# Risk factors for postpartum depression after cesarean section: a systematic review and meta-analysis

**DOI:** 10.7717/peerj.20550

**Published:** 2026-02-03

**Authors:** Yali Yu, Hui Feng, Peng Ma

**Affiliations:** 1 Beijing Anzhen Hospital Affiliated to Capital Medical University Nanchong Hospital, Nanchong Central Hospital, Nanchong, China; 2 The Second Clinical College of North Sichuan Medical College, Nanchong, China

**Keywords:** Postpartum depression, Risk factors, Cesarean section, Meta-analysis

## Abstract

**Background:**

The incidence of cesarean section (CS) is increasing each year and may be associated with an increased risk of postpartum depression (PPD). Although studies have examined the association between cesarean delivery and postpartum depression, the associated risk factors have not been fully investigated. This study aims to identify key risk factors for planned and emergency cesarean delivery through meta-analysis to help develop clinical prevention strategies.

**Methods:**

We searched multiple databases, including CNKI, Wan Fang, VIP, PubMed, Embase, Scopus, Google Scholar, CINAHL, Cochrane Library, and Web of Science, for studies published from the inception of these databases through January 8, 2025. The quality of the studies was assessed using the Newcastle-Ottawa Scale (NOS), and standardized mean difference (SMD) and ratio of ratios (OR) were used to assess the strength of association between different risk factors and postpartum depression, when I^2^ > 50%, a random effects model was used for data analysis; when I^2^ < 50%, a fixed effects model was chosen for analysis.

**Result:**

Nine articles (*n* = 3,338) were included in this study, meta-analysis results suggest that younger women (SMD = −0.16, 95% CI [−0.29 to −0.04], I^2^ = 0%), higher antenatal depression scores (SMD = 0.28, 95% CI [0.13–0.44], I^2^ = 15.1%), higher antenatal anxiety scores (SMD = 0.56, 95% CI [0.40–0.72], I^2^ = 35.6%) are more likely to experience postpartum depression, nulliparous (OR = 1.9, 95% CI [1.39–2.60], I^2^ = 0%) and elementary education level (OR = 1.34, 95% CI [1.05–1.72], I^2^ = 44.3%) were risk factor for postpartum depression after caesarean section.

**Conclusion:**

In summary, age, first-time pregnancy status, education level, and antenatal depression and anxiety scores are important risk factors for postpartum depression following cesarean delivery. Identifying and addressing these factors could provide valuable insights for the prevention and management of postpartum depression.

## Background

In recent years, the significant increase in the cesarean section rate has become a global concern. According to a report by the World Health Organization (WHO), the cesarean section rate has been steadily rising in low-middle-income countries, with some regions reaching or exceeding 30% (Parasiliti et al., 2023). This trend is potentially linked to the higher incidence of postpartum depression (PPD), a common mental health issue in the postpartum period that significantly affects the psychological and physical health of new mothers (Dennis et al., 2024; Moore Simas, Whelan & Byatt, 2023). PPD not only impacts a mother’s mood and quality of life but also has negative consequences for the infant’s growth and development, family relationships, and the broader social environment (Khadka et al., 2024), as the results of La Rosa, Alparone & Commodari (2025) suggest that PPD affects the quality of the mother-infant relationship. As a widely used method of delivery, cesarean section may increase the risk of maternal depression compared to natural delivery due to its unique procedure and extended recovery period (Evenson et al., 2024). However, cesarean sections can be categorized into elective (scheduled) and emergency procedures, with each potentially contributing to different psychological outcomes. Elective cesarean sections are planned, often for medical or personal reasons, while emergency cesarean sections occur when unforeseen complications arise during labor. Research suggests that the psychological impact of these two types of cesarean deliveries may differ. Elective CS may provide mothers with more time to prepare for the birth and recovery, potentially reducing feelings of anxiety and uncertainty. In contrast, emergency CS, due to its unexpected nature and often heightened medical complications, might result in greater emotional distress, potentially increasing the risk of postpartum depression (Wen & Wei, 2025).

Moreover, infection risks around the peripartum period, including surgical site infections, endometritis, or urinary tract infections, can extend the recovery process, necessitate further medical interventions, and worsen the overall experience of post-cesarean recovery. The prolonged recovery and potential for chronic pain can contribute to feelings of helplessness and anxiety, further increasing the risk of postpartum depression. Moreover, the high incidence of cesarean sections, combined with the unique challenges of the postoperative recovery period, can increase the likelihood of emotional distress and feelings of isolation among mothers (Munk-Olsen et al., 2023). The physical sequelae of cesarean delivery, such as scarring, changes in body appearance, and potential disfigurement, can also affect a mother’s self-esteem and body image. The visible scars from the surgery, especially when combined with post-operative pain or complications, may lead to psychological distress. Women may feel alienated from their pre-pregnancy identity or experience dissatisfaction with their physical appearance, which can exacerbate the emotional impact of the postpartum period (Hymas & Girard, 2019; Sheng et al., 2023). Although many studies have established a connection between cesarean delivery and an increased risk of postpartum depression, the specific risk factors and underlying mechanisms remain inadequately understood (Li et al., 2024; Shi et al., 2025).

In recent years, there has been a gradual increase in the number of studies addressing the risk factors associated with the development of postpartum depression in women after cesarean delivery, and some results have been achieved (Solé et al., 2020). Existing studies generally agree that prenatal anxiety, low social support, marital status, postnatal health problems, and psychological stress in post-caesarean delivery mothers are possible risk factors. In addition, an individual’s ability to regulate emotions, expectations of childbirth, and the socio-cultural environment are also important factors affecting maternal mental health (Gastaldon et al., 2022; Wen & Wei, 2025). To better understand these risk factors and to provide a basis for actual clinical interventions, many scholars have used methods such as systematic evaluation and meta-analysis to summaries and analyze the existing literature, with a view to revealing the key factors of postnatal depression in women after caesarean section (Ning et al., 2024).

Despite progress in this area, existing studies on postpartum depression following cesarean delivery have yielded inconsistent results (Orehek et al., 2012). Some research suggests that factors such as individual maternal characteristics, cultural background, and social support play a significant role in the development of depression, while other studies emphasize the influence of physiological factors and the specific nature of the cesarean procedure itself (Chen et al., 2024; Zhao & Zhang, 2020). These conflicting findings indicate potential limitations in the sample size, study design, and research methods of current studies. Therefore, it is crucial to conduct a systematic evaluation of existing research to better identify the true risk factors for postpartum depression following cesarean delivery.

The aim of this study was to comprehensively summarize the risk factors for postpartum depression following cesarean section through a meta-analysis. By screening and analyzing relevant literature, we aim to clearly identify the main risk factors associated with post-cesarean depression, offering a more scientific tool for clinical staff to predict and manage these risks. Additionally, this study will examine regional and cultural differences, providing insights for the development of more personalized psychological intervention strategies. By exploring the multifaceted factors contributing to maternal depression after cesarean delivery, this research seeks to promote early prevention and treatment, ultimately improving the overall health and well-being of both mothers and infants.

## Methods

This systematic evaluation and meta-analysis will strictly follow the Preferred Reporting Items for Systematic Reviews and Meta-Analyses (PRISMA) guidelines (Page et al., 2021). It is registered in Prospero with registration number CRD42025632540.

### Literature search

Computer searches were conducted in databases including CNKI, Wan fang, VIP, PubMed, Embase, Scopus, Google Scholar, CINAHL, Cochrane Library, and Web of Science. The study was registered with prospero on January 7, 2025, the search deadline was January 8, 2025 (As the literature was updated during this revision process, the cut-off date for the search is August 27, 2025), the raw data extraction was completed on January 20, 2025, and the analysis was completed on January 22, 2025, to ensure the comprehensiveness and accuracy of the literature review. To ensure comprehensiveness and accuracy, search terms included “postpartum depression”, “cesarean section”, and “risk factors”, and were combined using Boolean operators. See Table S1 for the specific search strategy and detailed information, there were no language restrictions in this meta-analysis.

### Literature screening

The current study used two authors to independently screen the literature for included studies using endnote 20. The inclusion criteria were (1) the inclusion population was pregnant women who met the criteria for cesarean section, (2) the case was postpartum depression (defined as Edinburgh Postnatal Depression Scale (EPDS) ≥10), the control was non-postpartum depression, and (3) the outcome indicator was a risk factor, the (4) the type of study was a cohort study.

Exclusion criteria for this study included (1) non-maternal groups or men; (2) those with a history of serious physical or mental illness during pregnancy or postpartum; (3) those with substance abuse behaviors such as drug and alcohol abuse; (4) those with serious postpartum complications such as postpartum hemorrhage, postpartum infections, (5) those who did not complete the EPDS screening after delivery or who were unable to provide data on the subject.

### Data extractions

Two authors—either a trained medical librarian with expertise in systematic literature retrieval or an obstetrician with clinical experience relevant to cesarean section research—independently reviewed the literature according to the predefined inclusion and exclusion criteria. In cases where discrepancies arose regarding study eligibility, the reviewers first attempted to resolve the disagreement through discussion. If consensus could not be reached, a third investigator adjudicated the final decision. For studies in which full texts were unavailable or essential data were missing, we contacted the corresponding authors. Articles were included only when complete full-text information and raw data were provided. Extracted data included the first author’s name, publication year, country, sample size, sex distribution, mean age, and diagnostic criteria. To ensure consistency in study selection and data extraction, inter-reviewer agreement was assessed using Cohen’s kappa statistic, yielding a value of 0.85, which reflects strong agreement. During the screening and extraction processes, we also examined the reference lists of included studies to ensure completeness and accuracy. All citations were cross-checked manually to confirm that no key literature was omitted and that all references adhered to relevant publication standards. All references were updated and verified accordingly. To ensure a comprehensive literature review, abstracts and unpublished studies were also considered. For abstracts, only those with sufficient data to assess study eligibility were included. If a study was unpublished but provided key data (through conference proceedings or clinical trial registries), we contacted the authors for additional information. Unpublished studies without available data were excluded.

### Quality evaluation

The quality of this included study was assessed using the Newcastle-Ottawa Scale (NOS) (Stang, 2010) for included studies. The NOS score is a commonly used tool specifically designed to assess the quality of non-randomized controlled studies such as cohort and case-control studies. The scale consists of three main sections: study selection, comparability and outcome assessment. In the Selection section, NOS assesses the representativeness of the study sample and the reasonableness of the inclusion criteria; in the Comparability section, it examines whether potential confounders have been considered and controlled for; and in the Outcome assessment section, it assesses the reliability of the results of the study and the accuracy of the methodology used to assess them. Each section has a corresponding scale with a total score of 9, with higher scores indicating higher study quality. By using the NOS score, this study was able to systematically assess the quality of the included literature and ensure the reliability and accuracy of the Meta-analysis results.

### Grade assessment

To systematically assess the quality of the evidence included in the study, this research used the Grading of Recommendations Assessment, Development, and Evaluation (GRADING) (Guyatt et al., 2008) system to rate the final evidence. The GRADING system is a tool widely used in clinical research that comprehensively evaluates the quality of evidence by assessing factors such as study design, risk of bias, consistency, directness, and precision. According to the GRADING system, evidence quality is divided into four levels: high, moderate, low, and very low.

### Data analysis

The combined effect sizes will be reported as ORs and their 95% CIs to allow for interpretation of results and statistical inference. Standardized mean difference (SMD) between the postpartum depression group and the non-postpartum depression group and 95% confidence interval (CI) were used for continuous variables and odds ratio (OR) and 95% CI for dichotomous variables. The heterogeneity of the model was evaluated using the I^2^ statistic. And I^2^ value above 50% indicated substantial heterogeneity, suggesting the need for further investigation into its sources. In cases of high heterogeneity, sensitivity analyses may be conducted to identify potential factors that could influence the pooled effect sizes. Asymmetry in the funnel plot indicates a higher likelihood of publication bias, which will be further evaluated using Egger’s test. *P* value < 0.05 suggests the presence of publication bias, while a *P* value > 0.05 suggests otherwise. If necessary, the trim-and-fill method will be used for further confirmation. In addition, random effects model and Egger’s test were used to assess publication bias.

## Result

### Literature screening results

**Figure 1:**
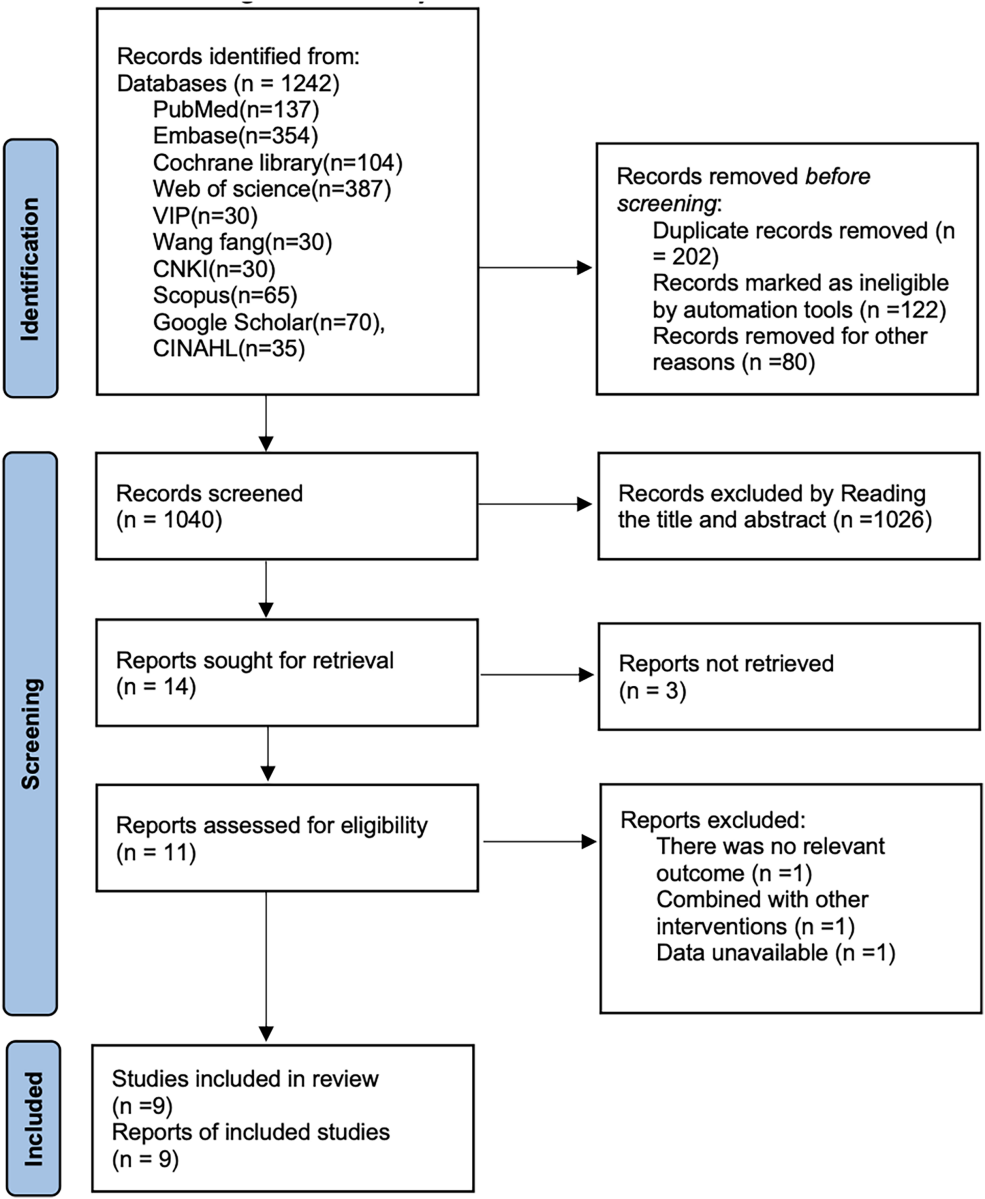
Literature search flow chart.

After searching CNKI (*n* = 30), Wan fang (*n* = 30), VIP Database (*n* = 30), PubMed (*n* = 137), Embase (*n* = 354), Cochrane Library (*n* = 104), Scopus (*n* = 65), Google Scholar (*n* = 70), CINAHL (*n* = 35) and Web of Science (*n* = 387), a total of 1,242 articles were retrieved. After removing 202 articles, 1,026 articles were excluded based on the title and abstract. After reading the full text, three more articles were excluded. Ultimately, nine cohort studies (Chandrasekaran et al., 2018; Lin et al., 2022; Quan et al., 2020; Shen et al., 2020; Tan et al., 2024; Wang, Liang & Su, 2024; Liu, 2021; Li, 2019; Hu, Hu & Zhang, 2022) were included. The literature search process is shown in Fig. 1.

### Basic characteristics

Nine cohort study articles (*n* = 3,338) were included in this study, six (Lin et al., 2022; Quan et al., 2020; Wang, Liang & Su, 2024; Liu, 2021; Li, 2019; Hu, Hu & Zhang, 2022) of which were from China, one (Chandrasekaran et al., 2018) from Canada, one (Shen et al., 2020) from Japan, and one (Tan et al., 2024) from Singapore, eight of which scored postpartum depression using an EPDS ≥10, and one (Quan et al., 2020) study using an EPDS ≥12. The basic characteristics are shown in Table 1.

**Table 1:** Table of basic characteristics.

Study	Year	Country	Study design	Sample size	Mean age
Chandrasekaran	2018	Canada	Cohort study	103	34.4
Lin	2022	China	Cohort study	590	32
Quan	2020	China	Cohort study	360	35
Tan	2024	Singapore	Cohort study	180	32.2
Wang	2024	China	Cohort study	287	32.4
Shen	2020	Japan	Cohort study	247	35
Liu	2021	China	Cohort study	80	37.42
Li	2019	China	Cohort study	1,238	34
Hu	2022	China	Cohort study	253	35

**Note:**

The diagnostic criteria for all studies were based on EPDS ≥10 (except Quan et al., 2020, where EPDS ≥12) (Chandrasekaran et al., 2018; Lin et al., 2022; Quan et al., 2020; Shen et al., 2020; Tan et al., 2024; Wang, Liang & Su, 2024; Liu, 2021; Li, 2019; Hu, Hu & Zhang, 2022).

### Quality evaluation

The quality assessment of this study is shown in Table 2, with three articles (Quan et al., 2020; Li, 2019; Hu, Hu & Zhang, 2022) scoring 7, three articles (Lin et al., 2022; Tan et al., 2024; Wang, Liang & Su, 2024) scoring 9, and the remaining articles scoring 8, the studies included in this study were of high quality. The results of the grade rating scale (Table 3), which was used in this study, suggested that all outcomes were of low quality.

**Table 2:** Newcastle-Ottawa Scale (NOS) scores.

Study	Representativeness of the exposed group	Selection of non-exposed groups	Determination of exposure factors	Identification of outcome indicators not yet to be observed at study entry	Comparability of exposed and unexposed groups considered in design and statistical analysis	Design and statistical analysis	Adequacy of the study’s evaluation of the outcome	Adequacy of follow-up in exposed and unexposed groups	Total scores
Chandrasekaran et al. (2018)	*	*	*	*	*	*	*	*	8
Lin et al. (2022)	*	*	*	*	**	*	*	*	9
Quan et al. (2020)	*	*	*	*	/	*	*	*	7
Tan et al. (2024)	*	*	*	*	**	*	*	*	9
Wang, Liang & Su (2024)	*	*	*	*	**	*	*	*	9
Shen et al. (2020)	*	*	*	*	*	*	*	*	8
Liu (2021)	*	*	*	*	*	*	*	*	8
Li (2019)	*	*	*	*	/	*	*	*	7
Hu, Hu & Zhang (2022)	*	*	*	*	/	*	*	*	7

**Note:**

Asterisks (*) indicate one score. Asterisks (**) indicate two score.

Denotes freely marked in NOS table by two investigators.

**Table 3:** Grade outcomes.

Outcomes	Grade
Age	Low
Nulliparous	Low
Primary education level	Low
Antenatal depression score	Low
Antenatal depression score	Low

### Meta-analysis results

#### Age

**Figure 2:**
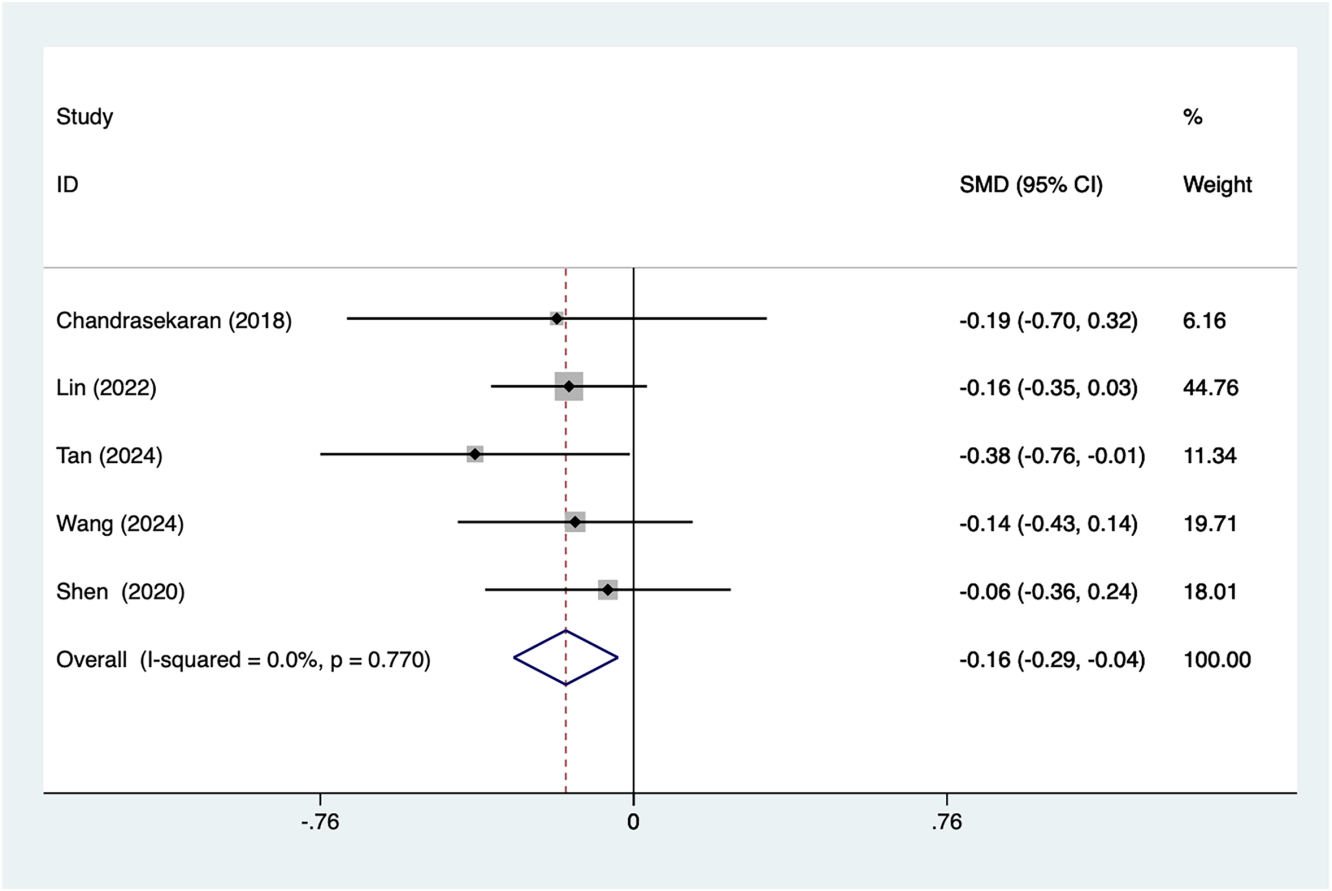
Forest plot shows the difference in age between the group with postpartum depression and the group without postpartum depression after cesarean section (Chandrasekaran et al., 2018; Lin et al., 2022; Shen et al., 2020; Tan et al., 2024; Wang, Liang & Su, 2024).

A total of five articles mentioned age as a risk factor. After conducting a heterogeneity test (I^2^ = 0%, *P* = 0.77), a fixed-effects model was used for analysis. The results (Fig. 2) suggest that an association between younger women and postpartum depression after caesarean section (SMD = −0.16, 95% CI [−0.29 to −0.04]).

#### Nulliparous

**Figure 3:**
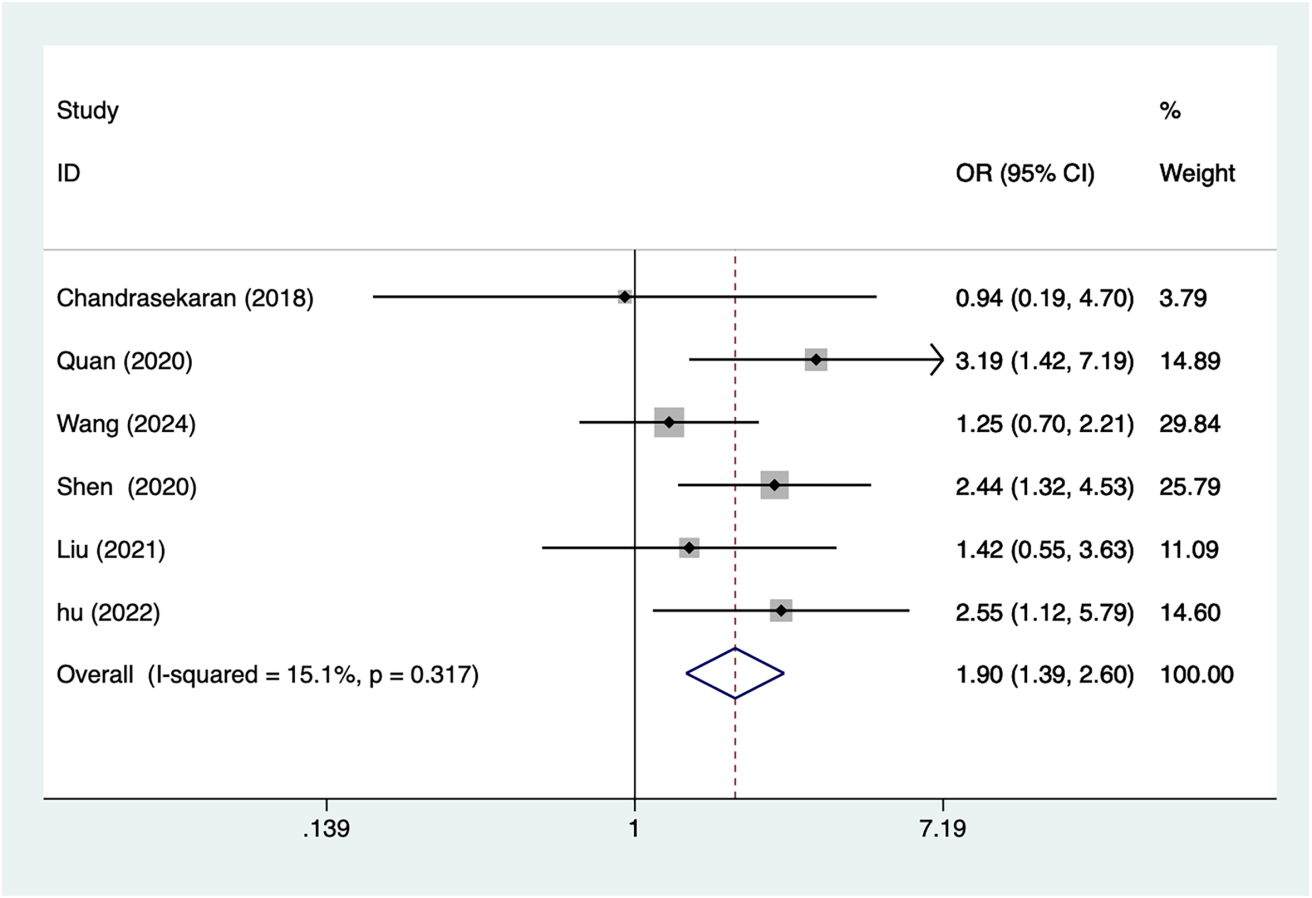
Forest plot of nulliparous was risk factors for postpartum depression after cesarean section by meta-analysis (Chandrasekaran et al., 2018; Quan et al., 2020; Shen et al., 2020; Wang, Liang & Su, 2024; Liu, 2021; Hu, Hu & Zhang, 2022).

A total of six articles mentioned nulliparous, which were analyzed by a fixed-effects model using a heterogeneity test (I^2^ = 15.1%, *P* = 0.317), and the results of the analysis (Fig. 3) suggested an association between nulliparity and postpartum depression after caesarean section (OR = 1.9, 95% CI [1.39–2.60]).

#### Elementary education level

**Figure 4:**
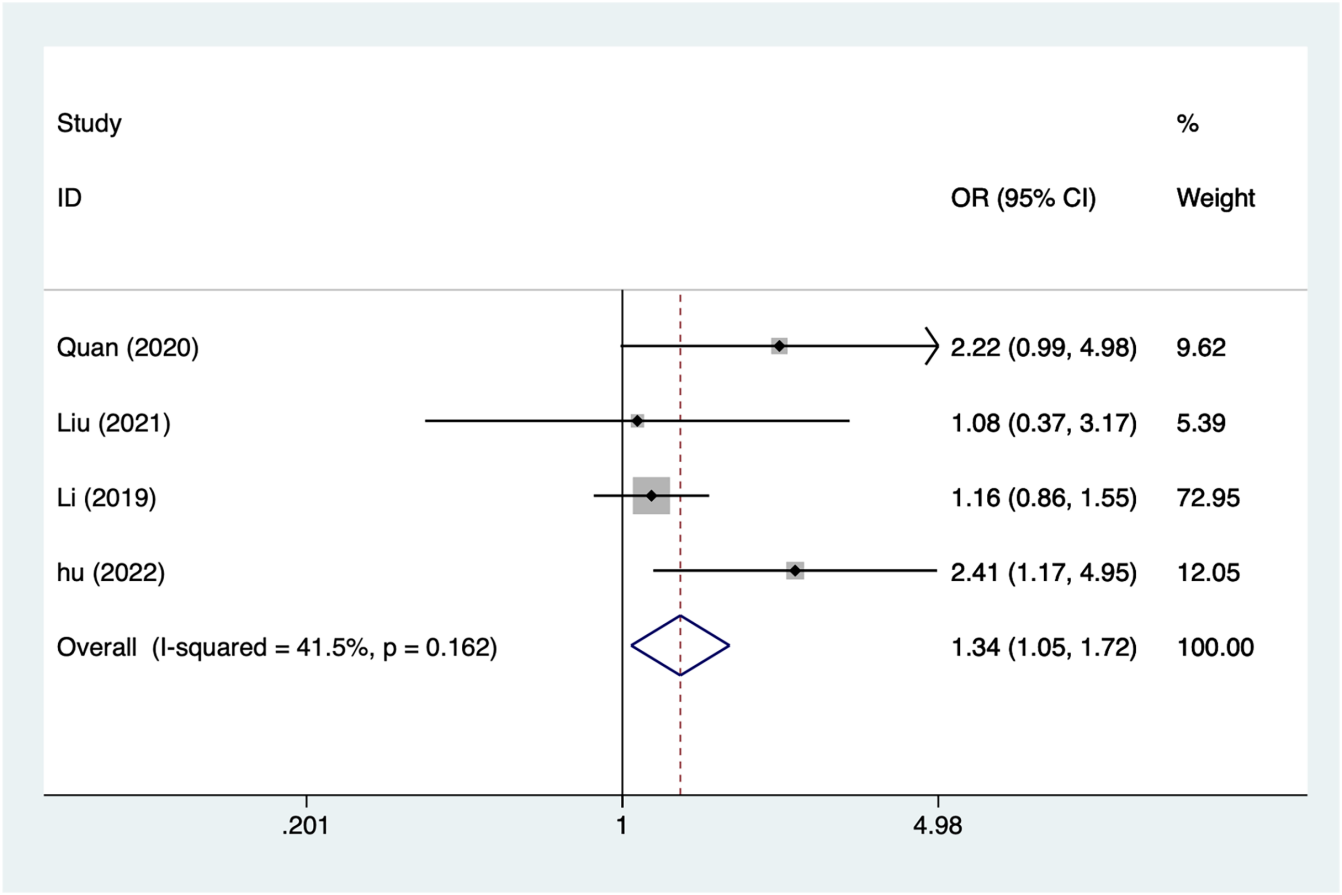
Forest plot of primary education level was risk factors for postpartum depression after cesarean section by meta-analysis (Quan et al., 2020; Liu, 2021; Li, 2019; Hu, Hu & Zhang, 2022).

A total of four articles mentioned elementary education level, which were analyzed by a fixed-effects model using a heterogeneity test (I^2^ = 41.5%, *P* = 0.162), and the results of the analysis (Fig. 4) suggested an association elementary education level and postpartum depression after caesarean section (OR = 1.34, 95% CI [1.05–1.72]).

#### Antenatal depression score

**Figure 5:**
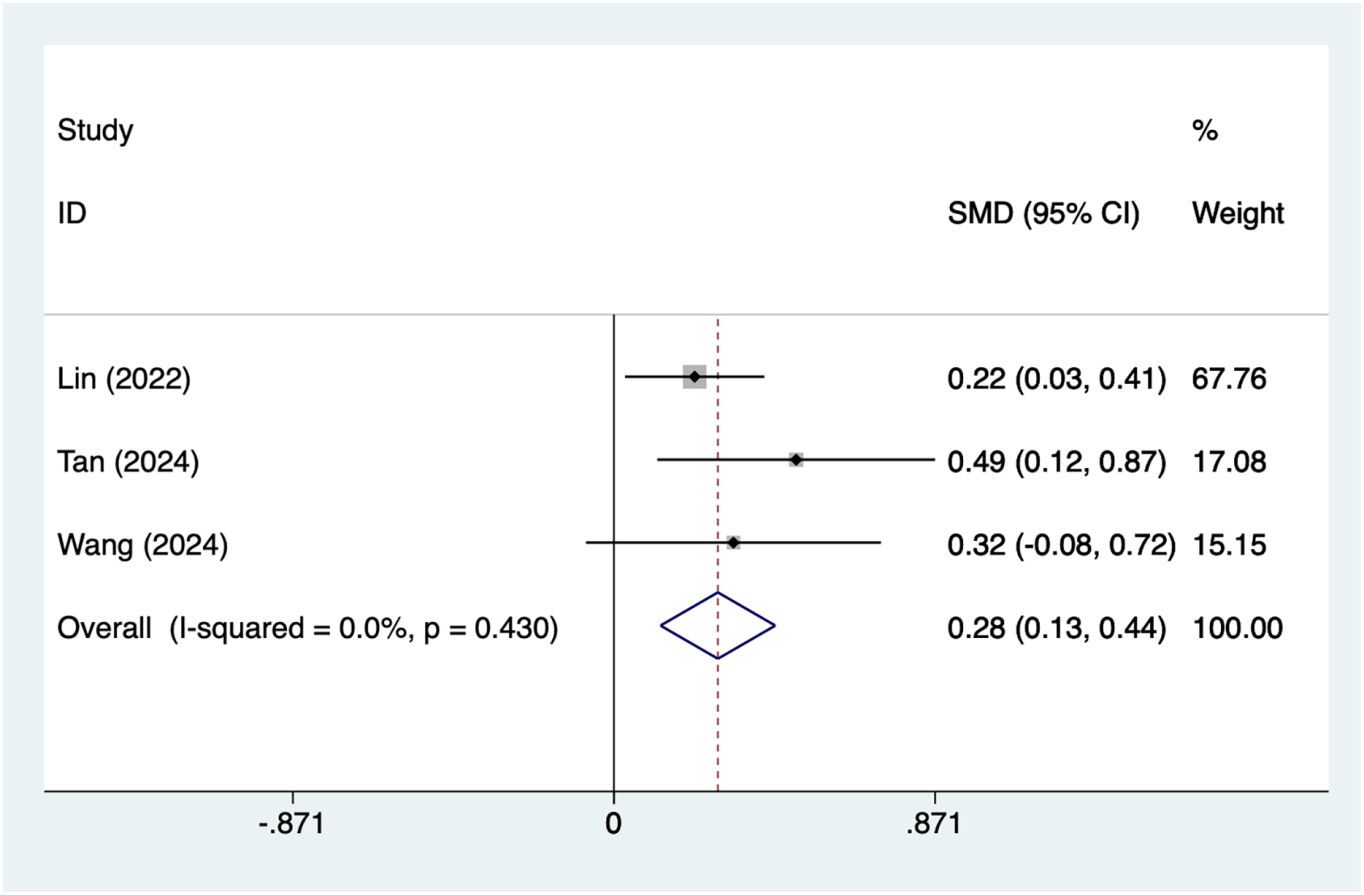
Forest plot of antenatal depression score was risk factors for postpartum depression after cesarean section by meta-analysis (Lin et al., 2022; Tan et al., 2024; Wang, Liang & Su, 2024).

A total of three articles mentioned antenatal depression score as a risk factor. After conducting a heterogeneity test (I^2^ = 0%, *P* = 0.43), a fixed-effects model was used for analysis. The results (Fig. 5) suggest an association higher antenatal depression scores and postpartum depression after caesarean section (SMD = 0.28, 95% CI [0.13–0.44]).

#### Antenatal depression score

**Figure 6:**
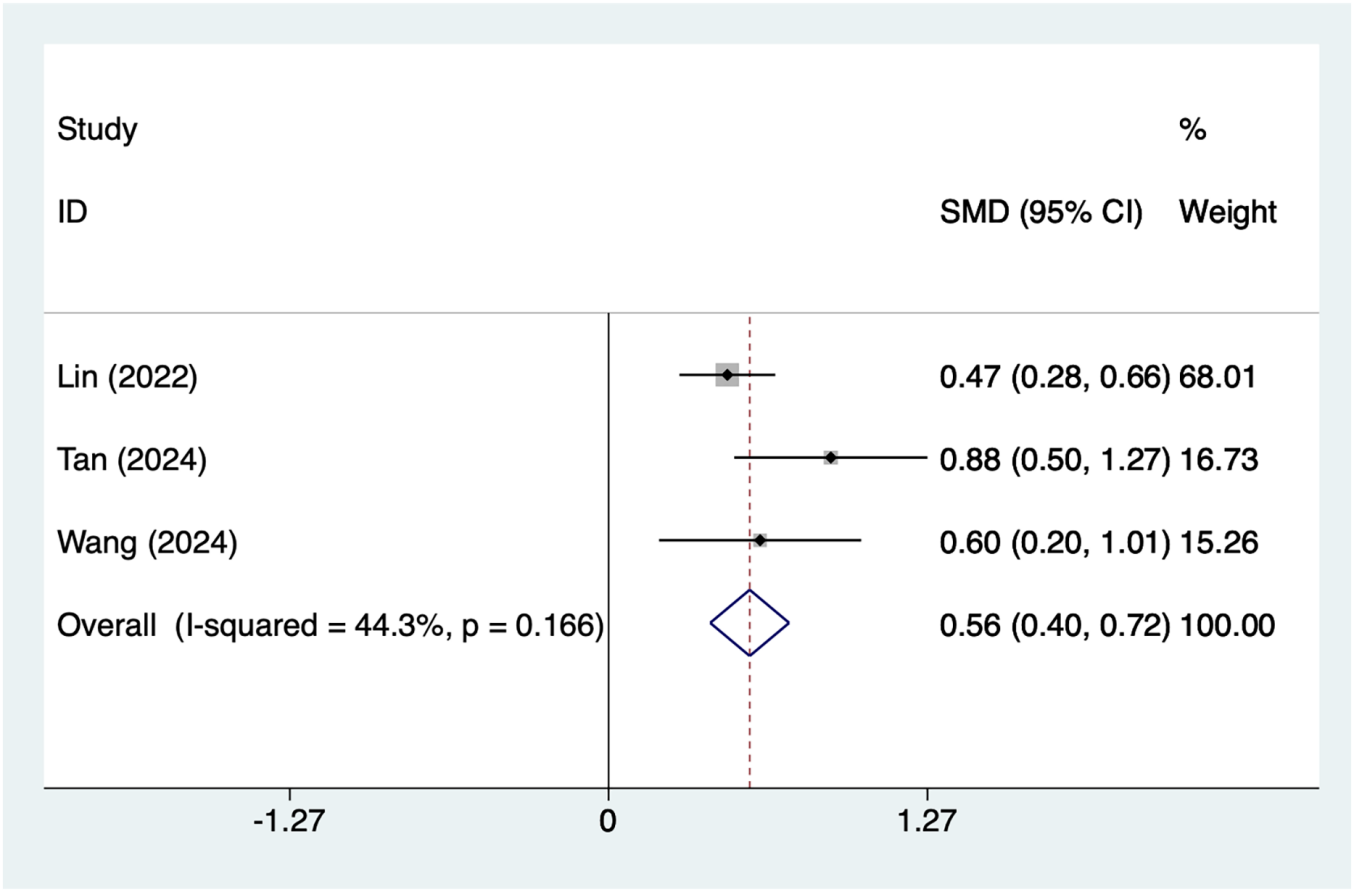
Forest plot of antenatal anxiety score was risk factors for postpartum depression after cesarean section by meta-analysis (Lin et al., 2022; Tan et al., 2024; Wang, Liang & Su, 2024).

A total of three articles mentioned antenatal anxiety score as a risk factor. After conducting a heterogeneity test (I^2^ = 44.3%, *P* = 0.166), a fixed-effects model was used for analysis. The results (Fig. 6) suggest an association woman with higher antenatal anxiety scores and postpartum depression after caesarean section (SMD = 0.56, 95% CI [0.40–0.72]).

#### Publication bias results

The current study used funnel plot and egger test to detect publication bias and the results of the study, which egger test age (Fig. S1) (*P* = 0.571), nulliparous (Fig. S2) (*P* = 0.861), elementary education level (Fig. S3) (*P* = 0.183), antenatal depression score (Fig. S4) (*P* = 0.354), antenatal anxiety score (Fig. S5) (*P* = 0.385), suggesting no publication bias. Funnel plots were visually inspected, and no asymmetry was observed, further supporting the absence of publication bias in our analysis. These findings suggest that the studies included in our meta-analysis were not significantly affected by publication bias, which adds credibility to the robustness of our conclusions.

## Discussion

The primary aim of this meta-analysis was to explore the risk factors associated with postpartum depression following cesarean delivery. The importance of this study lies in its ability to synthesize existing evidence and provide a comprehensive understanding of the factors that increase the likelihood of postpartum depression, which is critical for improving maternal mental health outcomes, and the results of the study concluded that the younger the age, the higher the prenatal anxiety and depression scores, nulliparous, and the more likely that cesarean pregnant women with primary school education are to develop postpartum depression.

The results of the current study suggest that younger women are more likely to develop postnatal depression after cesarean delivery. Younger women may face higher levels of psychological stress, especially if they lack parenting experience and have less social support (Batchelor et al., 2023; Petrosyan, Armenian & Arzoumanian, 2011). They may be more likely to feel physical and psychological discomfort, which may be exacerbated after delivery, leading to postpartum depression. For example, the Oztora et al. (2019) study found that younger mothers had significantly higher rates of postpartum depression than mothers of other ages. Therefore, it is important to provide psychological support and education to young mothers, especially during the antenatal and early postnatal period. Provide screening and intervention for postnatal depression to help them better adjust their psychological state and increase their confidence in parenting. Primiparas (first-time mothers) are a significant risk factor for postpartum depression. They face greater uncertainty and stress, particularly during the recovery process, often lacking accurate expectations about postpartum recovery (Škodová et al., 2022). New mothers frequently encounter multiple challenges, such as nighttime feeding and changes in body shape, which can increase their psychological burden (Vaillancourt et al., 2022). Similar studies have shown that the incidence of postpartum depression is significantly higher in primiparas compared to multiparas (Liu et al., 2017), difficulty adjusting to their new role can lead to increased anxiety and depressive symptoms. Therefore, new mothers should receive regular mental health assessment and counselling, especially during the antenatal and early postnatal periods. The risk of postnatal depression can be reduced by helping them to better adapt to their new roles through improved parenting knowledge, the establishment of support groups and the provision of adequate social support. There is a significant association between lower levels of education (especially primary school and below) and postpartum depression. Women with elementary education levels may lack awareness of the symptoms of postpartum depression and have difficulty accessing relevant mental health resources (Murakami et al., 2021). Their economic status and social support networks may be weaker, which exposes them to additional life stressors (Tsai et al., 2023). This result is in line with many studies, for example, Matsumura et al. (2019). also noted that low educational level is positively associated with the occurrence of postpartum depression, especially in low-income groups. Therefore, there is a need to strengthen health education for women with elementary education levels, especially on the identification of and ways to cope with postpartum depression. Provide more accessible mental health resources, such as free or low-cost psychosocial support services, especially by setting up special psychological counselling services in community health-care facilities or obstetrics outpatient clinics. Women with higher prenatal anxiety-depression scores are more likely to experience postpartum depression. Prenatal depression reflects mood disorders that may have already existed during a woman’s pregnancy, and these problems may be further exacerbated in the postpartum period (Witcraft et al., 2024). Research has shown that prenatal depression not only affects the mental health of the pregnant woman, but may also affect the development of the fetus, and that postnatal depression symptoms are more likely to be exacerbated by pre-existing depressive symptoms (Della Corte et al., 2022; Doty et al., 2022). Women with prenatal anxiety often have strong concerns about the labor process, recovery and parenting tasks, and these anxieties may continue into the postpartum period. Anxiety symptoms make them more sensitive to postpartum stress, leading to postpartum depression (Zhang et al., 2023). Many studies have also shown that prenatal anxiety and depression are closely related to postpartum depression. For example, Kaydırak et al. (2022) found that the severity of prenatal anxiety-depression symptoms significantly predicted the onset of postpartum depression. Therefore, anxiety management interventions such as cognitive behavioral therapy or relaxation training should be offered to pregnant women with high anxiety and depression. Prenatal anxiety screening and early intervention are particularly important, as well as group counselling and family support to alleviate pregnant women’s anxiety and help them better adapt to postnatal life.

In addition, the funnel plots corresponding to each of the above variables were symmetric, providing visual confirmation of the results from Egger’s test. A symmetric funnel plot typically indicates a lack of publication bias, suggesting that the studies included in our analysis are representative of the true effect sizes in the population. The absence of significant publication bias further strengthens the validity and reliability of the meta-analytic findings.

Given the small number of studies available for some risk factors, the null findings should be interpreted with great care. The absence of statistical significance for comorbidities during pregnancy, for example, may not reflect the true lack of association but could be a result of the limited data and insufficient power to detect significant effects. As the number of studies and the sample size increase, future research may yield different results. Therefore, while we have noted the null findings, we emphasize that further investigation with larger, more comprehensive datasets is necessary to assess the true relevance of these potential risk factors.

There are several limitations to this study that need to be acknowledged. Firstly, the sample size in this study was relatively small, which may have resulted in insufficient statistical power. This limited power could affect the reliability of the results, as evidenced by the failure of many of the risk factors in the original studies to reach statistical significance (*P* ≥ 0.05). This suggests that the current evidence may not be sufficient to establish clear associations between these factors and postpartum depression outcomes. Larger sample sizes and more robust study designs, such as longitudinal cohort studies or randomized controlled trials, should be considered in future research to provide stronger evidence.

Secondly, the risk factors analyzed in this study were assessed univariately without controlling for potential confounders. This methodological limitation means that the findings should be interpreted with caution. The lack of adjustment for confounding variables may have resulted in biased estimates of the associations between risk factors and postpartum depression. Future studies should employ multivariate analysis or other statistical techniques, such as propensity score matching, to control for confounders and provide more accurate and valid conclusions.

Finally, the overrepresentation of the Chinese sample is a limitation of this study. Due to the geographical limitation of the sample, the findings may not be fully generalizable to groups from other countries or cultural backgrounds. Therefore, future studies should consider including more diverse samples to improve the external validity of the results and to explore the risk factors for postpartum depression in different cultural contexts.

## Conclusion

In conclusion, age, primigravida status, education level, antenatal depression score, and antenatal anxiety score may be important risk factors for postpartum depression after cesarean delivery. The identification and intervention of these factors can provide important clues for the prevention and treatment of postpartum depression. Future studies should further explore other possible influencing factors, such as social support and cultural background, to gain a comprehensive understanding of the complex mechanisms underlying postnatal depression. Additionally, future research should consider studies that adjust for potential confounders and explore how confounder-adjusted separate studies can help clarify the causal relationships, providing a more robust basis for optimizing interventions.

## Supplemental Information

10.7717/peerj.20550/supp-1 Supplemental Information 1 Data extraction.

10.7717/peerj.20550/supp-2 Supplemental Information 2 Rationale.

10.7717/peerj.20550/supp-3 Supplemental Information 3 PRISMA checklist.

10.7717/peerj.20550/supp-4 Supplemental Information 4 Supplementary material.

## Additional Information and Declarations

### Data Availability

The following information was supplied regarding data availability:

This is a systematic review/meta-analysis.
