# Supplementary material for: Risk factors for postpartum depression after cesarean section: a systematic review and meta-analysis
Source: PeerJ. 2026 Feb 3;14:e20550. doi: 10.7717/peerj.20550 (PMC12880094; doi:10.7717/peerj.20550)
Supplement: Supplemental Information 2 [file peerj-14-20550-s002.docx]

• **Rationale for Conducting the Systematic Review/Meta-Analysis:** The rising rate of cesarean sections (C-sections) worldwide has been associated with various health outcomes, and postpartum depression (PPD) is one of the most significant mental health conditions that can occur after childbirth. Postpartum depression is a multifaceted disorder, and understanding the factors that increase its risk is critical for clinical practice. However, despite the growing body of research, there is no clear consensus on the key risk factors for postpartum depression specifically following cesarean delivery. Previous studies have identified individual risk factors, such as antenatal depression and anxiety, but many studies are limited by small sample sizes or lack of comprehensive meta-analytic approaches. By conducting this meta-analysis, the aim is to identify and quantify the risk factors for postpartum depression after cesarean delivery in a larger and more diverse sample of women, ultimately offering more robust evidence to guide clinical prevention and intervention efforts.

• **Contribution to Knowledge in Light of Previously Published Related Reports:** The findings from this meta-analysis contribute to the growing body of literature on postpartum depression following cesarean delivery by confirming and expanding on previous reports. For example, while other meta-analyses and studies have highlighted the role of antenatal depression and anxiety as predictors of postpartum depression, this analysis provides more specific and precise estimates of effect sizes and confidence intervals. The meta-analysis also introduces additional risk factors such as maternal age, primigravida status, and education level, which have been less frequently explored in previous studies. Notably, it emphasizes the importance of addressing these factors in the prenatal period, particularly for women at higher risk of postpartum depression. Furthermore, this meta-analysis supports the need for early identification and intervention in clinical settings to mitigate the negative outcomes of postpartum depression, particularly among women who undergo cesarean delivery. It builds on prior research by consolidating a larger body of evidence and offering a comprehensive risk profile that can be used for both clinical guidance and further research.
