# Supplementary material for: Risk factors for postpartum depression after cesarean section: a systematic review and meta-analysis
Source: PeerJ. 2026 Feb 3;14:e20550. doi: 10.7717/peerj.20550 (PMC12880094; doi:10.7717/peerj.20550)
Supplement: Supplemental Information 4 [file peerj-14-20550-s004.docx]

Table S1 Search strategy

((("Cesarean Section"[Mesh]) OR ((((((((((((Cesarean Section[Title/Abstract]) OR (Cesarean Sections[Title/Abstract])) OR (Abdominal Delivery[Title/Abstract])) OR (C-Section (OB[Title/Abstract]))) OR (C Section (OB[Title/Abstract]))) OR (C-Sections (OB[Title/Abstract]))) OR (Caesarean Section[Title/Abstract])) OR (Caesarean Sections[Title/Abstract])) OR (Delivery, Abdominal[Title/Abstract])) OR (Abdominal Deliveries[Title/Abstract])) OR (Deliveries, Abdominal[Title/Abstract])) OR (Postcesarean Section[Title/Abstract]))) AND (("Depression, Postpartum"[Mesh]) OR ((((((((((((((((((((Depression, Postpartum[Title/Abstract]) OR (Postpartum Depression[Title/Abstract])) OR (Post-Natal Depression[Title/Abstract])) OR (Depression, Post-Natal[Title/Abstract])) OR (Post Natal Depression[Title/Abstract])) OR (Post-Partum Depression[Title/Abstract])) OR (Depression, Post-Partum[Title/Abstract])) OR (Post Partum Depression[Title/Abstract])) OR (Postnatal Depression[Title/Abstract])) OR (Depression, Postnatal[Title/Abstract])) OR (Postnatal Dysphoria[Title/Abstract])) OR (Dysphoria, Postnatal[Title/Abstract])) OR (Postpartum Dysphoria[Title/Abstract])) OR (Dysphoria, Postpartum[Title/Abstract])) OR (Post-Partum Dysphoria[Title/Abstract])) OR (Dysphoria, Post-Partum[Title/Abstract])) OR (Post Partum Dysphoria[Title/Abstract])) OR (Post-Natal Dysphoria[Title/Abstract])) OR (Dysphoria, Post-Natal[Title/Abstract])) OR (Post Natal Dysphoria[Title/Abstract])))) AND (("Risk Factors"[Mesh]) OR (((((((((((((((((((Risk Factors[Title/Abstract]) OR (Factor, Risk[Title/Abstract])) OR (Risk Factor[Title/Abstract])) OR (Population at Risk[Title/Abstract])) OR (Populations at Risk[Title/Abstract])) OR (Risk Scores[Title/Abstract])) OR (Risk Score[Title/Abstract])) OR (Score, Risk[Title/Abstract])) OR (Risk Factor Scores[Title/Abstract])) OR (Risk Factor Score[Title/Abstract])) OR (Score, Risk Factor[Title/Abstract])) OR (Health Correlates[Title/Abstract])) OR (Correlates, Health[Title/Abstract])) OR (Social Risk Factors[Title/Abstract])) OR (Factor, Social Risk[Title/Abstract])) OR (Factors, Social Risk[Title/Abstract])) OR (Risk Factor, Social[Title/Abstract])) OR (Risk Factors, Social[Title/Abstract])) OR (Social Risk Factor[Title/Abstract])))


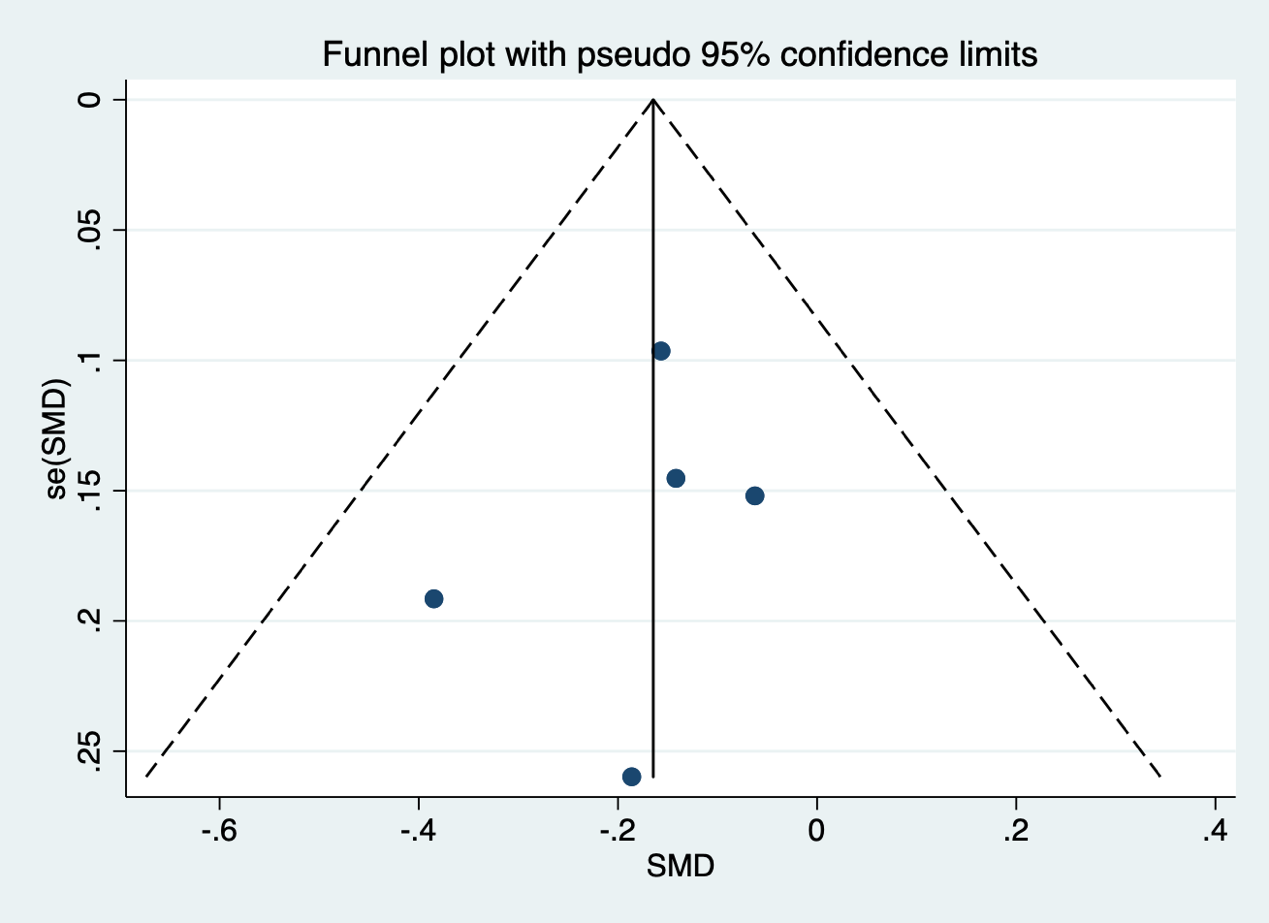


figure S1 Funnel plot of age


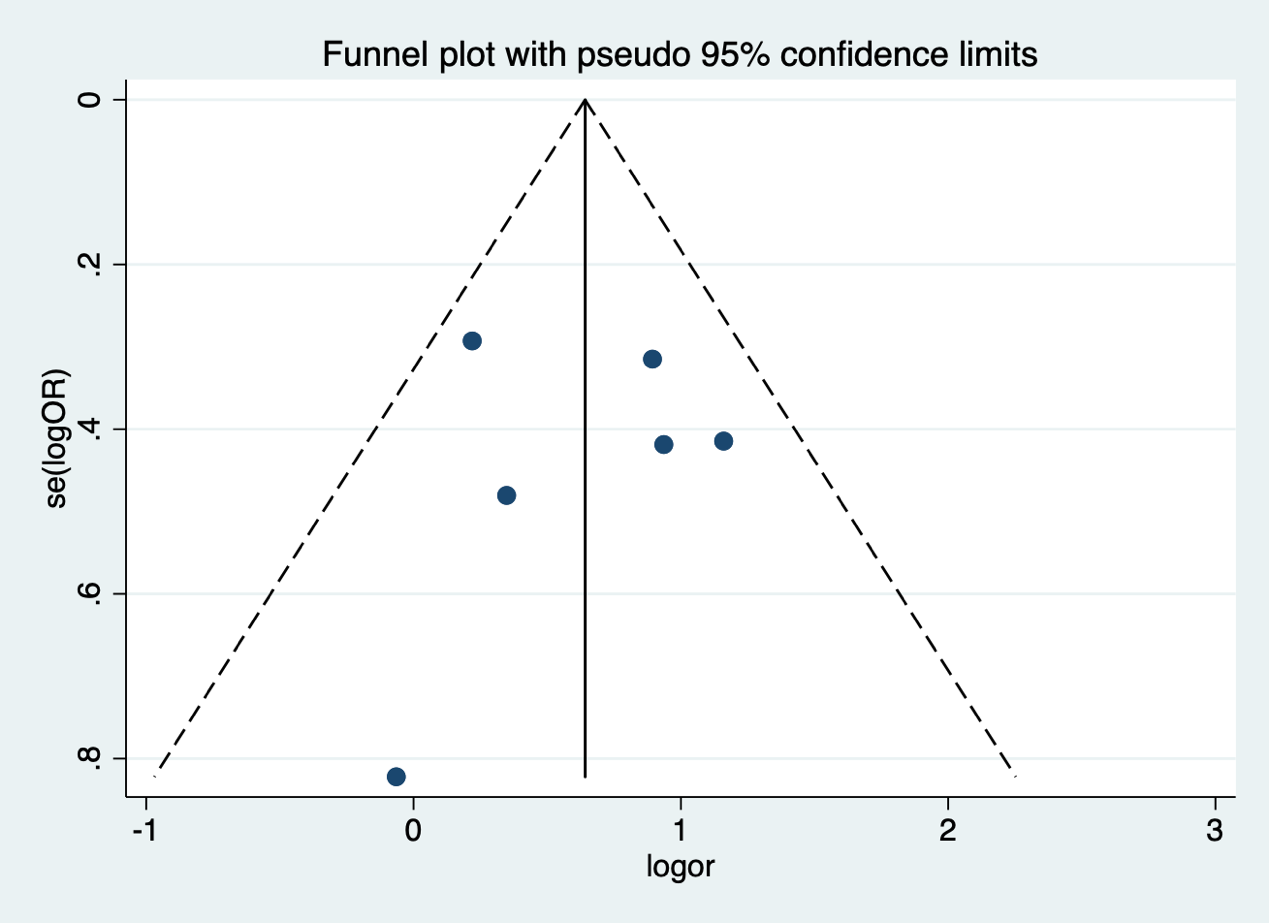


figure S2 Funnel plot of nulliparous


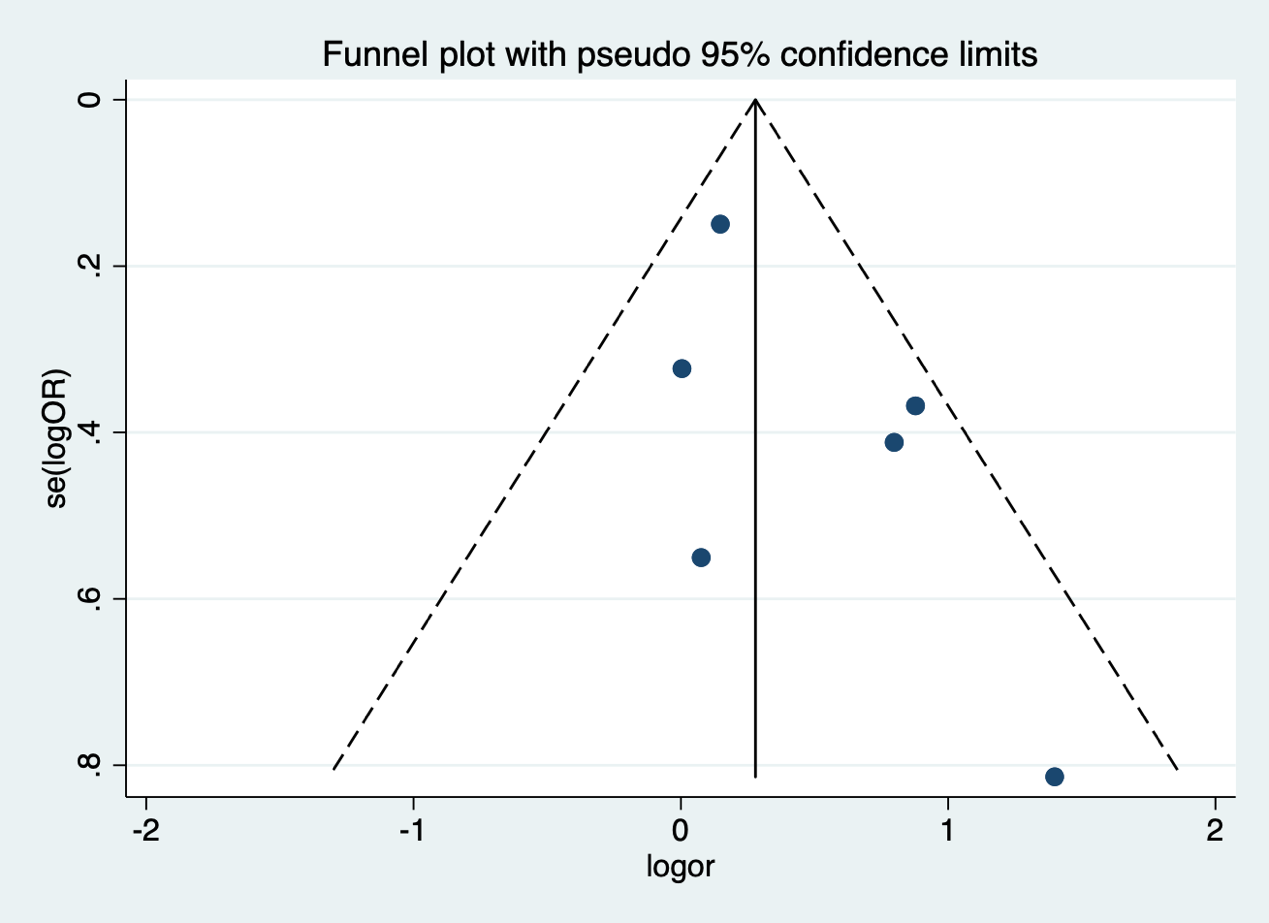


figure S3 Funnel plot of primary education level


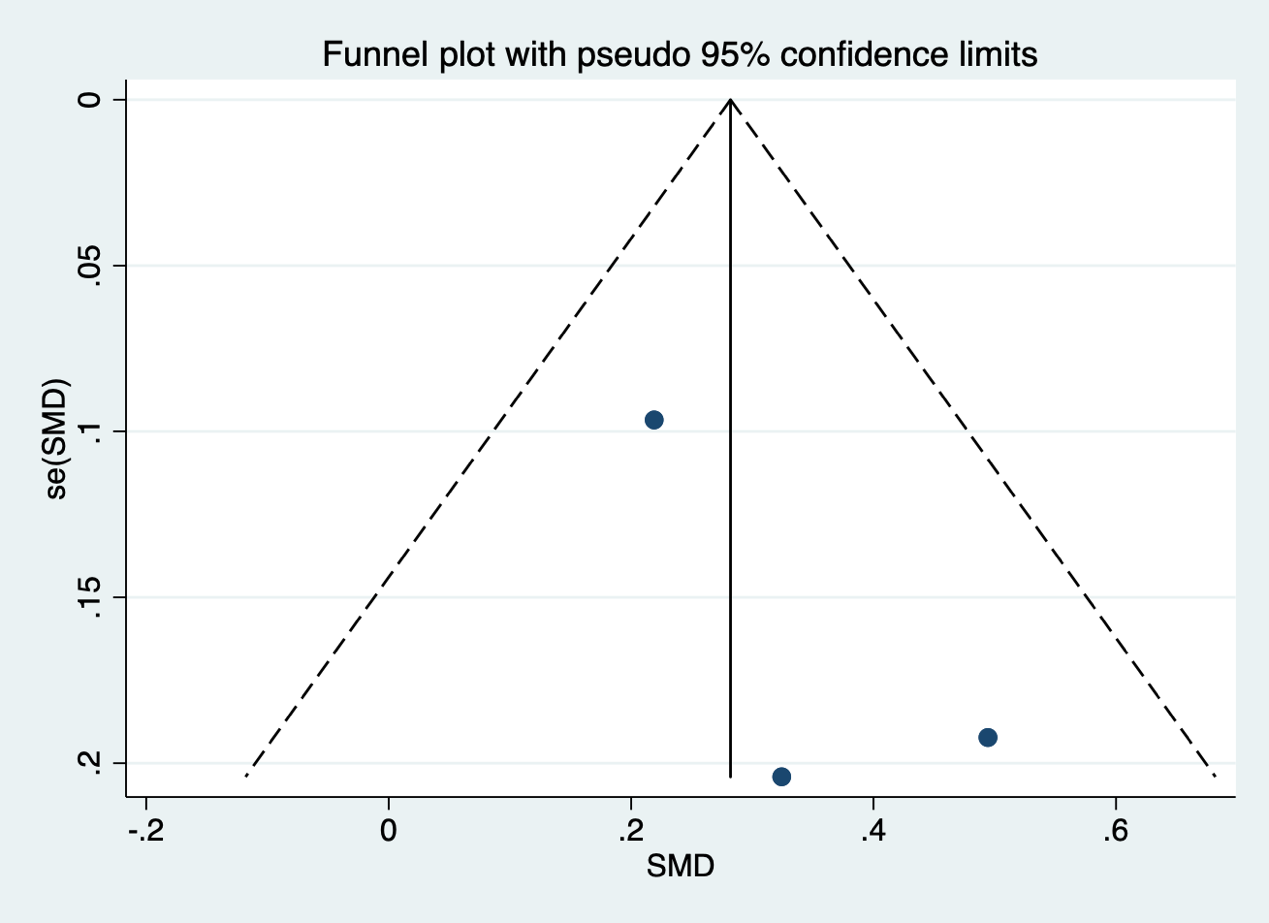


figure S4 Funnel plot of antenatal depression score


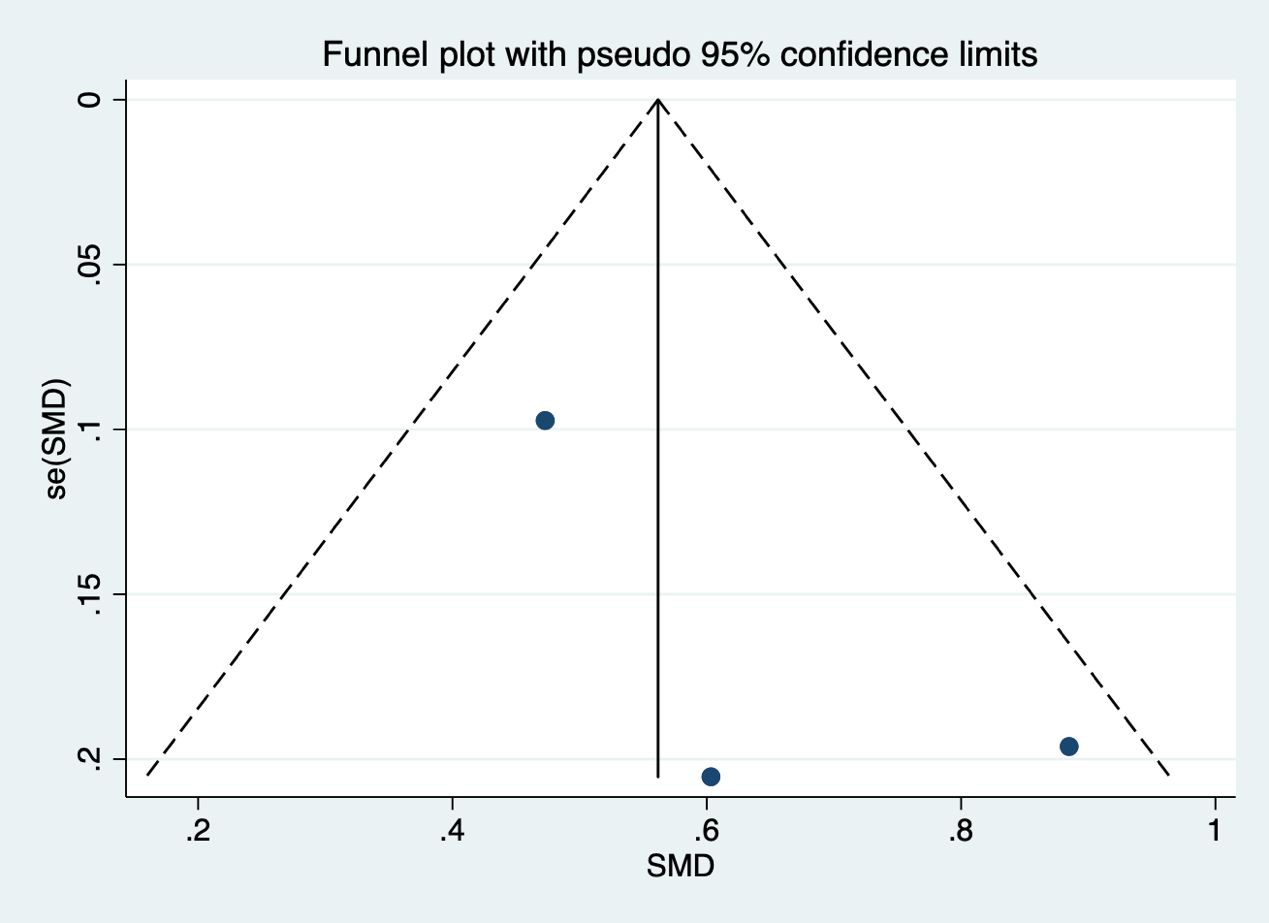


figure S5 Funnel plot of antenatal anxiety score
